# Influence of Knee Immobilization on Chondrocyte Apoptosis and Histological Features of the Anterior Cruciate Ligament Insertion and Articular Cartilage in Rabbits

**DOI:** 10.3390/ijms18020253

**Published:** 2017-01-26

**Authors:** Hirotaka Mutsuzaki, Hiromi Nakajima, Yasuyoshi Wadano, Syogo Furuhata, Masataka Sakane

**Affiliations:** 1 Department of Orthopaedic Surgery, Ibaraki Prefectural University of Health Sciences, 4669-2 Ami, Inashiki-gun, Ibaraki 300-0394, Japan; 2 Department of Agriculture, Ibaraki University, 3-21-1 Chuo, Ami, Ibaraki 300-0393, Japan; hiromi.nakajima.vmd@vc.ibaraki.ac.jp (H.N.); 15a112g@vc.ibaraki.ac.jp (S.F.); 3 Department of Rehabilitation Medicine, Ibaraki Prefectural University of Health Sciences, 4669-2 Ami, Inashiki-gun, Ibaraki 300-0394, Japan; wadano@ipu.ac.jp; 4 Department of Orthopaedic Surgery, Tsukuba Gakuen Hospital, 2573-1 Kamiyokoba, Tsukuba, Ibaraki 305-0854, Japan; sakane-m@tsukuba-seikei.jp

**Keywords:** anterior cruciate ligament insertion, articular cartilage, chondrocyte apoptosis, glycosaminoglycan, immobilization

## Abstract

This study examined the influence of immobilization on chondrocyte apoptosis and histological features of the anterior cruciate ligament (ACL) insertion and knee articular cartilage in rabbits. Forty-eight male Japanese white rabbits were assigned to an immobilization (*n* = 24) or sham (*n* = 24) group. Rabbits in the immobilization group underwent complete unilateral surgical knee immobilization and rabbits in the sham group underwent a sham surgery. The average thickness of the glycosaminoglycan (GAG) stained red area by safranin O staining, the chondrocyte apoptosis rate and the chondrocyte proliferation rate in the cartilage layer in the ACL insertion and the articular cartilage of the medial tibial condyle were measured at one, two, four and eight weeks in six animals from each group. In the ACL insertion, the chondrocyte apoptosis rate was higher in the immobilization group than in the sham group at two and eight weeks after surgery (*p* < 0.05). The chondrocyte proliferation rate gradually decreased from two weeks to eight weeks in the immobilization group. The GAG layer was thinner in the immobilization group than in the sham group at two, four and eight weeks after surgery (*p* < 0.05). In the articular cartilage, the chondrocyte apoptosis rate in the immobilization group was higher than in the sham group at four and eight weeks after surgery (*p* < 0.05). The GAG layer was significantly thinner in the immobilization group than that in the sham group at four and eight weeks after surgery (*p* < 0.05). Knee immobilization significantly increased chondrocyte apoptosis at two and eight weeks after surgery in the ACL insertion and at four and eight weeks after surgery in the articular cartilage of the medial tibial condyle, and decreased GAG layer thickness from two to eight weeks after surgery in the ACL insertion and from four to eight weeks after surgery in the articular cartilage.

## 1. Introduction

Tendon and ligament insertions, such as the insertion of the anterior cruciate ligament (ACL), include four transitional tissue layers: tendon/ligament, unmineralized fibrocartilage, mineralized fibrocartilage, and bone [1]. The different stiffnesses of these tissue layers reduce stress concentration in the insertion site [1]. Similarly, articular cartilage comprises four distinguishable zones, and this zonal arrangement also reduces stress concentration [2]. Glycosaminoglycans (GAGs) in the cartilage layer of ligamentous insertions and in the articular cartilage provide tissue hydration and elasticity [3,4]. The GAGs in the cartilage layer of tendon and ligament insertions mainly resist tensile and shear stresses, while GAGs in articular cartilage primarily resist compressive stress [1,2,3,4]. In both articular cartilage and tendon/ligament insertion sites, the GAG layer plays an important rule for load transmission. An understanding of the extracellular, matrix-derived structural properties of the ACL insertion and knee articular cartilage is necessary for early and appropriate management of injury, disuse, and degeneration.

We have previously reported an increased chondrocyte apoptosis rate and histological changes in the cartilage layers in the tibial insertions of human ACLs post-rupture [5,6]. An increased chondrocyte apoptosis rate also preceded a decrease in thickness of GAG layer in the ACL insertion in an ACL resection animal model [7,8]. In addition, we reported that mechanical unloading increased chondrocyte apoptosis, decreased chondrocyte proliferation, and decreased the GAG layer thickness of the patellar tendon (PT) insertion for up to six weeks in rabbits [9]. In these previous studies [5,6,7,8,9], joint motion was allowed.

Tendons degenerate when immobilized; the mean failure loads for Achilles’ tendons from rabbits immobilized for four and eight weeks were significantly smaller than those of tendons from control rabbits [10]. Ligament insertions may also degenerate over time after immobilization. In rats, articular cartilage thickness has been shown to decrease significantly in immobilized joints [11]. Similarly, an increase in apoptotic cells in the articular cartilage of rabbits after immobilization has also been demonstrated [12]. However, the influence of knee immobilization on chondrocyte apoptosis, chondrocyte proliferation, and GAG layer thickness in the ACL insertion and articular cartilage of the medial tibial condyle have not been reported. We hypothesized that immobilization would increase chondrocyte apoptosis and decrease chondrocyte proliferation in both the ACL insertion and the articular cartilage of the tibia. This study examined the influence of immobilization on histological features within the ACL insertion and articular cartilage of the medial tibial condyle in rabbits.

## 2. Results

### 2.1. Chondrocyte Apoptosis Rates, Determined Based on Terminal Deoxynucleotidyl Transferase-Mediated Deoxyuridine Triphosphate-Biotin Nick-End Labeling (TUNEL)-Positive Chondrocytes

**Figure 1:**
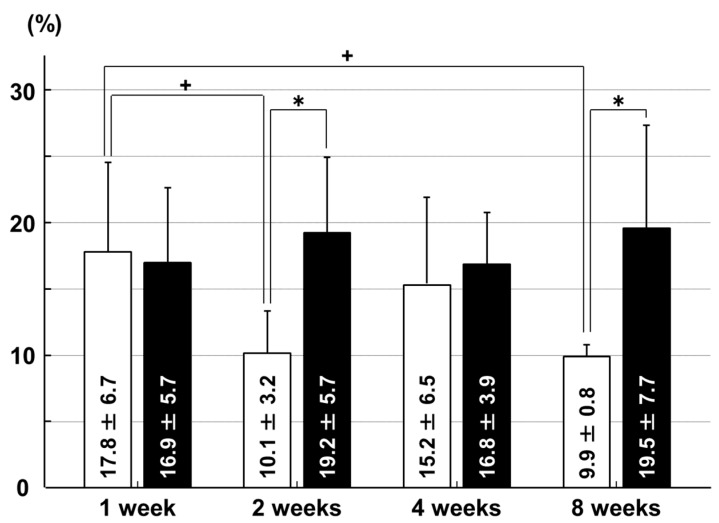
Chondrocyte apoptosis rates in the anterior cruciate ligament (ACL) insertion. □: sham group; ■: immobilization group. * *p* < 0.05; + *p* < 0.05; *n* = 6.

In the ACL insertion, the chondrocyte apoptosis rate was higher in the immobilization group than in the sham group at two weeks (*p* = 0.028) and eight weeks (*p* = 0.029) after surgery. In the sham group, the apoptosis rate at one week was higher than at two weeks (*p* = 0.030) and eight weeks (*p* = 0.017; Figure 1).

**Figure 2:**
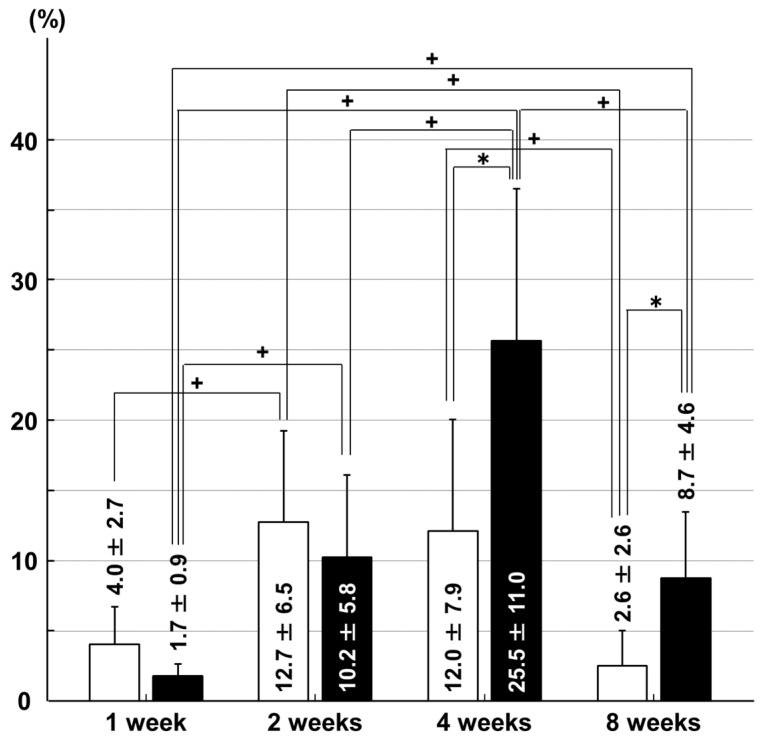
Chondrocyte apoptosis rates in the articular cartilage of the medial tibial condyle. □: sham group; ■: immobilization group. * *p* < 0.05; + *p* < 0.05; *n* = 6.

In the tibial articular cartilage, the chondrocyte apoptosis rate was higher in the immobilization group than in the sham group at four weeks (*p* = 0.035) and eight weeks (*p* = 0.019) after surgery. In the immobilization group, the apoptosis rate at four weeks was higher than that at one week (*p* = 0.003), two weeks (*p* = 0.013) and eight weeks (*p* = 0.011). The apoptosis rate at two weeks and eight weeks was higher than that at one week (*p* = 0.017 and *p* = 0.017, respectively) in the immobilization group. In the sham group, the apoptosis rate at two weeks was higher than that at one week (*p* = 0.019) and eight weeks (*p* = 0.010). The apoptosis rate at four weeks was higher than at that eight weeks (*p* = 0.034) in the sham group (Figure 2).

### 2.2. Chondrocyte Proliferation Rates, Determined by Proliferating Cell Nuclear Antigen (PCNA)-Positive Chondrocytes

**Figure 3:**
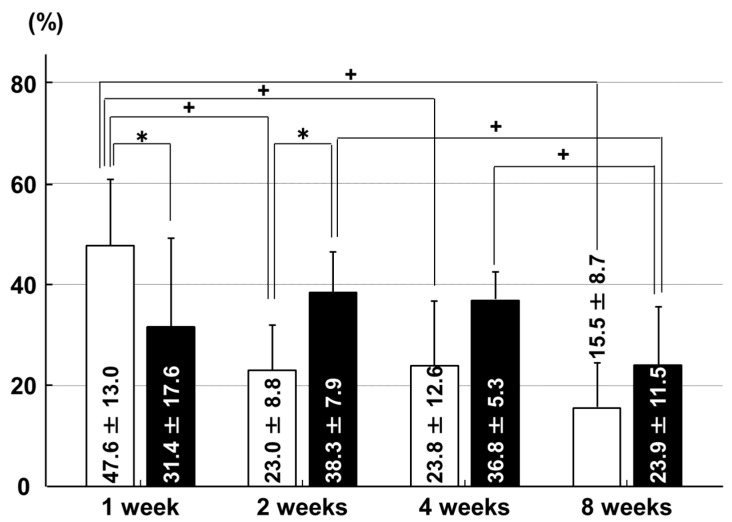
Chondrocyte proliferation rates in the ACL insertion. □: sham group; ■: immobilization group. * *p* < 0.05; + *p* < 0.05; *n* = 6.

In the ACL insertion, the chondrocyte proliferation rate was higher in the sham group than in the immobilization group at one week after surgery (*p* = 0.006). This had reversed by two weeks after surgery, when the chondrocyte proliferation rate was higher in the immobilization group than in the sham group (*p* = 0.010). In the immobilization group, the chondrocyte proliferation rate at eight weeks was lower than that at two weeks (*p* = 0.031) and four weeks (*p* = 0.032). In the sham group, the chondrocyte proliferation rate at one week was higher than that at two weeks (*p* = 0.003), four weeks (*p* = 0.009) and eight weeks (*p* < 0.001; Figure 3).

**Figure 4:**
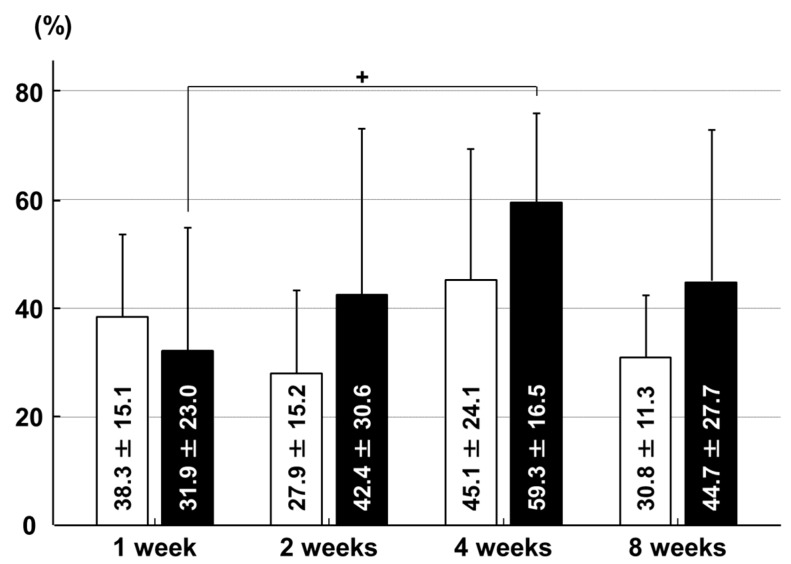
Chondrocyte proliferation rates in the articular cartilage of the medial tibial condyle. □: sham group; ■: immobilization group. + *p* < 0.05; *n* = 6.

In the articular cartilage of the medial tibial condyle, there were no significant between-group differences in the chondrocyte proliferation rate at any time point. In the immobilization group, the chondrocyte proliferation rate at four weeks was higher than that at one week (*p* = 0.039; Figure 4).

### 2.3. Thicknesses of Glycosaminoglycan (GAG)-Stained Areas

**Figure 5:**
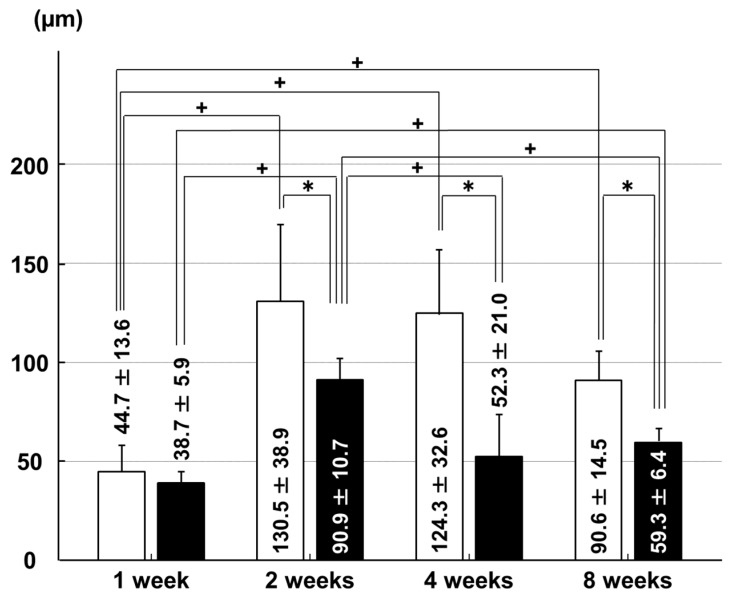
Thicknesses of Glycosaminoglycan (GAG)-stained areas in the ACL insertion. □: sham group; ■: immobilization group. * *p* < 0.05; + *p* < 0.05; *n* = 6.

**Figure 6:**
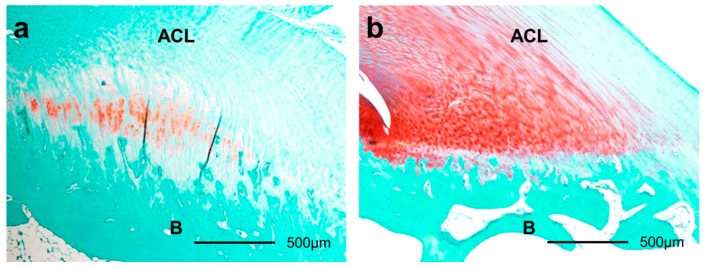
Histological sections of ACL insertions eight weeks after surgery by safranin O staining (40×). The smaller GAG-stained red area in immobilization group (**a**) than that in the sham group (**b**) was shown. ACL: anterior cruciate ligament. B: bone.

The GAG-stained areas in the ACL insertion were thinner in the immobilization group than that in the sham group at two weeks (*p* = 0.048), four weeks (*p* = 0.001), and eight weeks (*p* < 0.001) after surgery. In the immobilization group, the GAG-stained areas were thicker at two weeks than they were at one week (*p* < 0.001), four weeks (*p* = 0.002), and eight weeks (*p* < 0.001), and the GAG-stained areas were thinner at one week than they were at two weeks (*p* < 0.001) and eight weeks (*p* < 0.001). In the sham group, the GAG-stained areas were thinner at one week than they were at two weeks (*p* < 0.001), four weeks (*p* < 0.001), and eight weeks (*p* = 0.008; Figure 5 and Figure 6).

**Figure 7:**
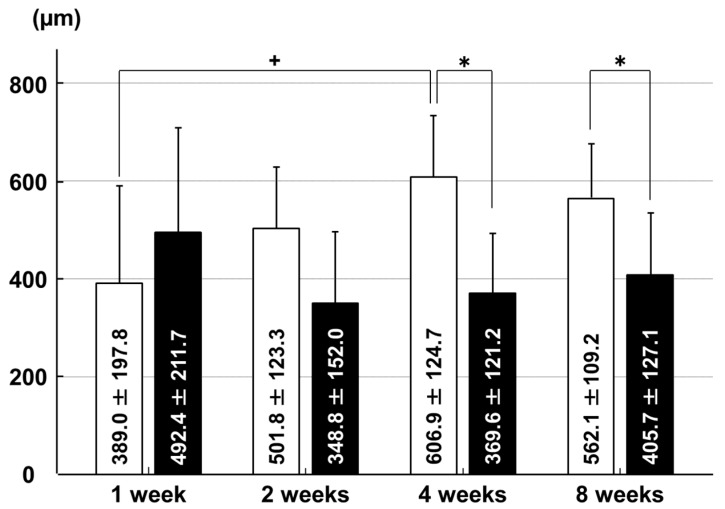
Thicknesses of GAG-stained areas in the articular cartilage of the medial tibial condyle. □: sham group; ■: immobilization group. * *p* < 0.05; + *p* < 0.05; *n* = 6.

**Figure 8:**
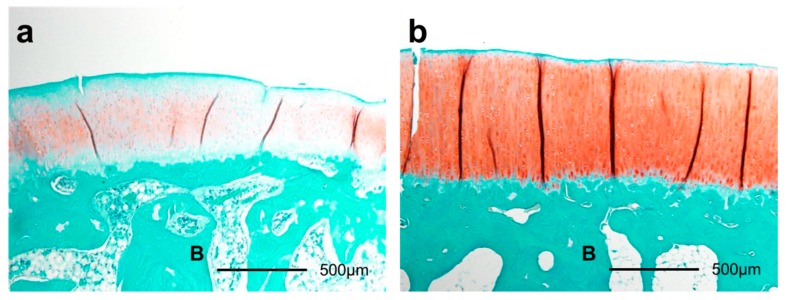
Histological sections of articular cartilage eight weeks after surgery by safranin O staining (40×). The smaller GAG-stained red area in the immobilization group (**a**) than that in the sham group (**b**) was shown. B: bone.

The GAG-stained areas in the articular cartilage of the medial tibial condyle were thinner in the immobilization group than in the sham group at four weeks (*p* = 0.007) and eight weeks (*p* = 0.045). In the sham group, the GAG-stained areas were thinner at one week than at four weeks (*p* = 0.046; Figure 7 and Figure 8).

## 3. Discussion

Immobilization significantly increased chondrocyte apoptosis from two to eight weeks after surgery in the ACL insertion and the tibial articular cartilage, leading to a reduction in GAG thickness in the ACL insertion and tibial articular cartilage for up to eight weeks post-operatively.

In the present study, the apoptosis rates in the ACL insertion and tibial articular cartilage were higher in the immobilization group than in the sham group at two and eight weeks and at four and eight weeks after surgery, respectively. These histological changes are similar to our previous results in the ACL resection animal model [7,8], and in the PT insertion after mechanical unloading [9]. The chondrocyte apoptosis rates in the ACL resection and in the mechanical unloading were significantly higher than in the sham group from one to four weeks after surgery [7,8,9]. The mechanical environment experienced by the ACL insertion and articular cartilage in an immobilized knee may resemble that after ligament resection or mechanical unloading, with very little load being placed on the insertion of the ligament or the articular cartilage. However, the rabbits in the present study were allowed to bear weight on the immobilized limb. Therefore, the mechanical stress on the ACL insertion and knee articular cartilage in the present study may have been greater than the mechanical stress after ACL resection or mechanical unloading of the PT. This may explain the later start of chondrocyte apoptosis in the present study compared with that seen after ACL resection or mechanical unloading of the PT in our previous studies. However, despite this delay, immobilization did significantly increase chondrocyte apoptosis in the ACL insertion and the knee articular cartilage. In normal adult rats and mice, 3% to 10% of the cells are apoptotic in intact articular cartilage [13]; a similar percentage of apoptotic chondrocytes in the intact ACL insertion has also been shown in immature rabbits [8]. Increased chondrocyte apoptosis can lead to imbalances in the concentrations of matrix metalloproteinases and their inhibitors, and in other proinflammatory cytokines [14,15,16]. In the acute and subacute periods after injury, inflammatory cytokines likely regulate chondrocyte apoptosis and matrix degradation [17,18]. In addition, mechanical factors can directly affect apoptosis in tissues [19,20,21]. The chondrocyte apoptosis rate at one week was higher than that at two weeks and eight weeks in the ACL insertion in the sham group. Such reactions may occur in association with the inflammatory response after surgery.

In the present study, there were no significant between-group differences in the tibial articular cartilage chondrocyte proliferation rates at any time point. There were also no significant between-group differences in chondrocyte proliferation rates in the ACL insertion at four and eight weeks after surgery. This differs from the findings of our previous report, in which the chondrocyte proliferation rates in the mechanical unloading group were lower than in the sham group at four and six weeks post-operatively [9], suggesting that mechanical unloading decreased chondrocyte proliferation. Although mechanical unloading completely removed stress from the PT insertion, immobilization still allowed weight bearing in the immobilized limbs. This difference in the mechanical environment in the current study versus that in our previous study [9] likely explains the disparity in our results; chondrocyte proliferation did not decrease in the present study because of the mechanical stresses caused by weight bearing. The cell proliferation rate at eight weeks was lower than that at two weeks and four weeks in the ACL insertion, whereas there were no significant differences between any time points in the tibial articular cartilage. The ACL insertion may receive less mechanical stress than the articular cartilage after immobilization. In the ACL insertion, the chondrocyte proliferation rate was higher in the sham group than in the immobilization group at one week, and was higher in the immobilization group than in the sham group at two weeks after surgery. This phenomenon may be due to the proinflammatory responses to the surgical insult at one week, and a positive feedback reaction to increased chondrocyte apoptosis at two weeks after surgery.

We also found that the GAG-stained areas were thinner in the immobilization group than in the sham group at two and eight weeks after surgery in the ACL insertion and at four and eight weeks after surgery in the tibial articular cartilage. Increased chondrocyte apoptosis led to a decrease in GAG layer thickness in the insertion site and the articular cartilage after immobilization. The chain of events observed in the present study was similar to that observed after ACL resection [5,6,7,8,9,22]. In both groups, the GAG layer thicknesses in the ACL insertion at one week were lower than at two, four, and eight weeks after surgery. These histological changes may be associated with the surgical insult and subsequent inflammation. The increase in chondrocyte apoptosis and subsequent decrease in GAG layer thickness occurred earlier in the ACL insertion than in the tibial articular cartilage. Because the cartilage layer is thinner and contains fewer chondrocytes in the ACL insertion than in the tibial articular cartilage, the effects of immobilization were first detectable in the ACL insertion. Another possible explanation for the difference in timing of the effects of immobilization is that the mechanical load experienced by the ACL insertion may have been smaller than that experienced by the tibial articular cartilage.

Clinically, the knowledge of the histological changes in the ACL insertion and adjacent articular cartilage after immobilization is important for determining the best approaches for rehabilitation of patients with ligament injuries, fracture, disuse atrophy, and degenerative joint decease. Our results suggest that knee immobilization should be limited to less than one month to minimize degeneration of the ACL insertion and the articular cartilage. Moreover, a detailed understanding of the injured or degenerative insertion and articular cartilage may help to determine the etiology, and may help in devising better treatment protocols for knee injuries that control and limit chondrocyte apoptosis.

Further immunohistochemical and mechanical analyses, and research into recovery after immobilization, are necessary to define the key factors that control the regeneration or degeneration of the ACL insertion and the adjacent articular cartilage. Additional immobilization experiments using an animal model with plaster cast fixation would complement this work.

## 4. Materials and Methods

### 4.1. Surgical Procedure

**Figure 9:**
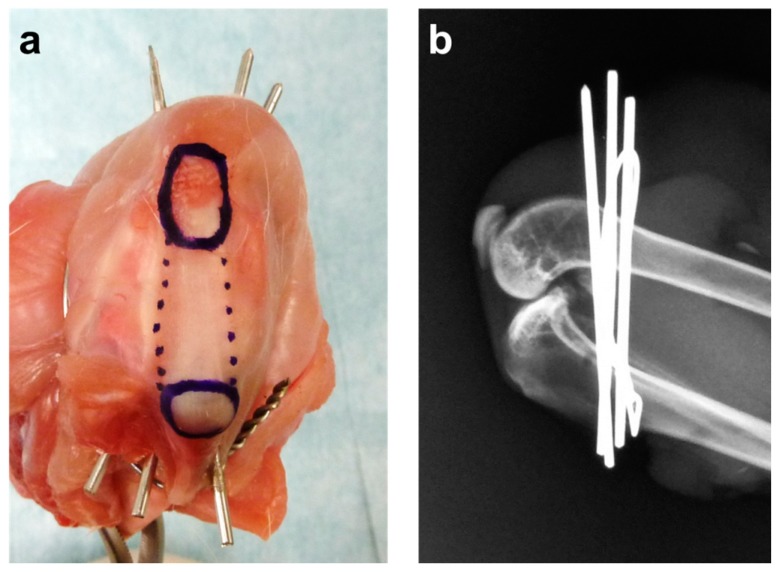
Right knee of a cadaver rabbit. (**a**) The knee was completely immobilized by three stainless steel wires installed between the distal femur and proximal tibia, and a soft stainless steel wire coiled between the distal femur and proximal tibia. The ends of the soft stainless steel wire were firmly anchored. The upper blue circle is the patella. The lower blue circle is the tibial tubercle. The region indicated by the dotted lines is the patellar tendon; (**b**) Lateral radiographs were taken to confirm that the wires were not within the knee joint.

Forty-eight skeletally immature male Japanese white rabbits (weight range: 2.5–3.0 kg) were used for this study. The rabbits were maintained in accordance with the guidelines of the Ethical Committee of the University of Tsukuba and the National Institutes of Health guidelines for the care and use of laboratory animals (NIH Pub. No. 86-23 Rev. 1985). The rabbits were randomly divided into an immobilization (*n* = 24) and a sham (*n* = 24) group. After intravenous barbiturate injection (40 mg/kg), an anterior skin incision was made over the right knee. The knee was completely immobilized in deep flexion with three stainless steel wires (1.6 mm diameter) installed between the distal femur and proximal tibia, and a soft stainless steel wire (0.8 mm diameter) coiled between the distal femur and proximal tibia with its ends firmly anchored. Complete immobilization in deep knee flexion was confirmed, preventing knee motion throughout the experimental period. This surgical method was simulated using a cadaver rabbit before the start of this study (Figure 9a), and we radiographically confirmed that the wires were not within the knee joint capsule (Figure 9b). In the sham group, we performed the same surgical procedure on animals, except the wires were removed immediately after installation. The incision was closed with a 2–0 non-absorbable suture. After surgery, the animals were allowed to move freely in their cages and did not receive antibiotics. Six animals in each group were sacrificed at 1, 2, 4 and 8 weeks after surgery.

### 4.2. Histomorphological Analysis

**Figure 10:**
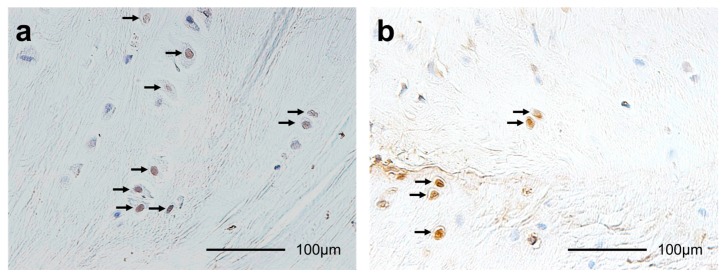
Histological sections of (**a**) terminal deoxynucleotidyl transferase-mediated deoxyuridine triphosphate-biotin nick-end labeling (TUNEL) staining (400×); TUNEL-positive chondrocytes are brown (arrows); and (**b**) proliferating cell nuclear antigen (PCNA) staining (400×); PCNA-positive chondrocytes are brown (arrows).

ACL-tibia complexes from each animal’s were fixed in 10% neutral-buffered formalin for 1 week. After fixation, all specimens were decalcified using 10% ethylenediaminetetraacetic acid (pH 7.4) and embedded in paraffin. The specimens were sliced to 5 mm thick in the center of the ACL insertion site and the center of the medial tibial condyle. These were stained with hematoxylin and eosin (HE) to assess histomorphology and with safranin O to assess GAG content [5,6,7,8,9]. In the ACL insertion and tibial articular cartilage, we performed terminal deoxynucleotidyl transferase-mediated deoxyuridine triphosphate-biotin nick-end labeling (TUNEL) for detecting apoptotic cells [5,6,7,8,9] (Figure 10a), and proliferating cell nuclear antigen (PCNA) for detecting proliferating cells [6,9] (Figure 10b).

We performed TUNEL staining according to the manufacturer’s instructions (Apoptag^®^ Plus Peroxidase In Situ Apoptosis Detection kit, Merck Millipore, Billerica, MA, USA). TUNEL-positive nuclei of the chondrocytes were stained dark brown, and TUNEL-negative nuclei of the chondrocytes were stained blue [5,6,7,8,9].

We performed PCNA immunostaining according to the manufacturer’s instructions (Histofine^®^ SAB-PO(M) kit, Nichirei Biosciences Inc., Tokyo, Japan). Sections were deparaffinized and rinsed in phosphate buffered saline (PBS) for 5 min. Next, they were immersed in 3% hydrogen peroxide (H_2_O_2_) in methanol for 10 min to block endogenous peroxidase activity. Afterwards, the slides were rinsed in PBS for 5 min, blocked in 10% normal rabbit serum at 25 °C for 10 min, and incubated at 4 °C for 12 h with a monoclonal antibody against PCNA at 1:100 dilution (PC-10, Code No. M0879, Dako, Glostrup, Denmark). We used Antibody Diluent (Code No. S0809, Dako) as the primary antibody for the negative controls [6,9].

The histomorphometric analysis was performed using a method similar to that used in our previous studies [5,6,7,8,9,22]. We examined the sections using a light microscope (BX-51, Olympus Optical Co., Ltd., Tokyo, Japan). The total cartilage layer area was identified by HE staining. We measured the GAG-stained red area by safranin O staining in the cartilage layer in the ACL insertion and the articular cartilage. Mac Scope software (Mitani Co., Fukii, Japan) was used to determine the total number of chondrocytes and the numbers of TUNEL-positive and PCNA-positive chondrocytes in the ACL insertion and the cartilage layer. Each GAG-stained red area was divided by the width of the ACL insertion or the articular cartilage to define the average thickness of the GAG-stained red areas. The TUNEL-positive and PCNA-positive rates were calculated by normalizing to the total number of chondrocytes in the cartilage layer.

### 4.3. Statistical Analysis

The histomorphometric data were compared within and between groups using Student’s *t*-tests. Differences with a *p* < 0.05 were considered statistically significant.

## 5. Conclusions

Knee immobilization significantly increased chondrocyte apoptosis in the ACL insertion at two and eight weeks after surgery and in the tibial articular cartilage at four and eight weeks after surgery. The GAG layer thickness decreased from two to eight weeks after surgery in the ACL insertion and from four to eight weeks after surgery in the tibial articular cartilage in rabbits. These results suggest that the duration of immobilization should be limited to less than one month to minimize degeneration of the ACL insertion and the articular cartilage.
